# Abdominal FLASH irradiation reduces radiation-induced gastrointestinal toxicity for the treatment of ovarian cancer in mice

**DOI:** 10.1038/s41598-020-78017-7

**Published:** 2020-12-10

**Authors:** Karen Levy, Suchitra Natarajan, Jinghui Wang, Stephanie Chow, Joshua T. Eggold, Phoebe E. Loo, Rakesh Manjappa, Stavros Melemenidis, Frederick M. Lartey, Emil Schüler, Lawrie Skinner, Marjan Rafat, Ryan Ko, Anna Kim, Duaa H. Al-Rawi, Rie von Eyben, Oliver Dorigo, Kerriann M. Casey, Edward E. Graves, Karl Bush, Amy S. Yu, Albert C. Koong, Peter G. Maxim, Billy W. Loo, Erinn B. Rankin

**Affiliations:** 1 Department of Radiation Oncology, Stanford University School of Medicine, Stanford, CA 94305 USA; 2 Department of Obstetrics and Gynecology, Stanford University School of Medicine, Stanford, CA 94305 USA; 3 Department of Comparative Medicine, Stanford University School of Medicine, Stanford, CA 94305 USA; 4 Stanford Cancer Institute, Stanford University School of Medicine, Stanford, CA 94305 USA; 5 Department of Chemical and Biomolecular Engineering, Vanderbilt University School of Engineering, Nashville, TN 37235 USA; 6 Division of Radiation Oncology, University of Texas MD Anderson Cancer Center, Houston, TX 77030 USA; 7 Department of Radiation Oncology, Indiana University School of Medicine, Indianapolis, IN 46202 USA

**Keywords:** Preclinical research, Translational research, Ovarian cancer

## Abstract

Radiation therapy is the most effective cytotoxic therapy for localized tumors. However, normal tissue toxicity limits the radiation dose and the curative potential of radiation therapy when treating larger target volumes. In particular, the highly radiosensitive intestine limits the use of radiation for patients with intra-abdominal tumors. In metastatic ovarian cancer, total abdominal irradiation (TAI) was used as an effective postsurgical adjuvant therapy in the management of abdominal metastases. However, TAI fell out of favor due to high toxicity of the intestine. Here we utilized an innovative preclinical irradiation platform to compare the safety and efficacy of TAI ultra-high dose rate FLASH irradiation to conventional dose rate (CONV) irradiation in mice. We demonstrate that single high dose TAI-FLASH produced less mortality from gastrointestinal syndrome, spared gut function and epithelial integrity, and spared cell death in crypt base columnar cells compared to TAI-CONV irradiation. Importantly, TAI-FLASH and TAI-CONV irradiation had similar efficacy in reducing tumor burden while improving intestinal function in a preclinical model of ovarian cancer metastasis. These findings suggest that FLASH irradiation may be an effective strategy to enhance the therapeutic index of abdominal radiotherapy, with potential application to metastatic ovarian cancer.

## Introduction

Radiation therapy (RT) is the most effective cytotoxic cancer therapy for the treatment of localized tumors and it is used to treat 60% of patients with cancer in the United States^1^. However, radiotherapy is still limited by toxicities to nearby normal tissues. For example, intestinal injury is the primary dose-limiting factor in RT for patients with abdominal and pelvic tumors. Approximately 60–80% of patients who receive large field pelvic or abdominal radiation therapy experience acute bowel toxicity symptoms including nausea, abdominal pain, diarrhea, and fatigue^2^. Moreover, patients can develop delayed radiation enteropathy, which is a chronic and progressive disease associated with significant long-term morbidity. In addition to reducing patient quality of life, radiation-induced injury to the intestine limits the radiation dose and curative potential of the therapy for many patients with abdominal and pelvic tumors including women with metastatic ovarian cancer^3–5^.

Ovarian cancer is the leading cause of gynecological cancer-related deaths among women in developed countries. Mortality rates are particularly high in ovarian cancer because the majority of women are diagnosed with advanced stage disease in which the tumor has disseminated beyond the ovaries and pelvic organs to the peritoneum and abdominal organs including the diaphragm, stomach, omentum, liver, and intestines. Historically, total abdominal irradiation (TAI) was used as an effective postsurgical adjuvant therapy in the management of metastatic ovarian cancer^6–8^. However, given the risk of gastrointestinal and hematopoietic toxicity associated with large field abdominal radiation, chemotherapy has been preferred over abdominal irradiation in the treatment of metastatic ovarian cancer^3–5^. More recently, there is renewed interest in using radiotherapy to increase the efficacy of chemotherapy, immunotherapy and targeted therapies in the treatment of ovarian cancer^9^. However, the gastrointestinal toxicities associated with abdominal irradiation are of significant concern. Therefore, new strategies are needed to enhance the therapeutic index of RT for the treatment of abdominal and pelvic tumors.

Ultra-high dose rate FLASH irradiation is emerging as a new strategy to reduce radiation-induced normal tissue toxicity and enhance the therapeutic index of radiation therapy. We and others have recently reported that ultra-high dose rate FLASH irradiation reduces radiation-induced toxicity to multiple normal tissues. In contrast to clinically used conventional dose rates of about 2–20 Gy/min (CONV), FLASH irradiation at dose rates of > 40 Gy/s (more than two orders of magnitude higher) produces less radiation-induced lung fibrosis and radiation-induced neurocognitive impairment after lung and brain irradiation, respectively^10–12^. FLASH mediated sparing of the skin from radiation-induced necrosis has been demonstrated in minipigs, cats, and mice^13,14^. Meanwhile, in preclinical models of lung cancer, FLASH achieves similar tumor control as CONV irradiation^10^. These findings demonstrated localized FLASH irradiation of the lung, skin, or brain could reduce radiation induced toxicities. However, ovarian cancer presents with widespread metastasis throughout the peritoneal cavity and abdomen requiring a wide abdominal irradiation field that includes the highly radiation sensitive intestine. A radiation treatment modality that could reduce gastrointestinal toxicities associated with total abdominal irradiation, while maintaining tumor control, could be transformative in the treatment of ovarian cancer.

Here we demonstrate that total abdominal FLASH irradiation reduces radiation-induced intestinal injury and preserves intestinal function in both healthy and tumor-bearing mice. Compared to conventional dose rate, FLASH irradiation reduces cell death in intestinal crypt base columnar cells (CBCs) and enhances crypt regeneration. Importantly, FLASH irradiation provides similar efficacy to CONV RT in controlling ovarian cancer peritoneal metastases. Thus, our findings identify a potential strategy to enhance the therapeutic index of abdominal irradiation for metastatic ovarian cancer.

## Results

### FLASH irradiation produces less lethality from radiation-induced gastrointestinal syndrome than CONV irradiation

**Figure 1:**
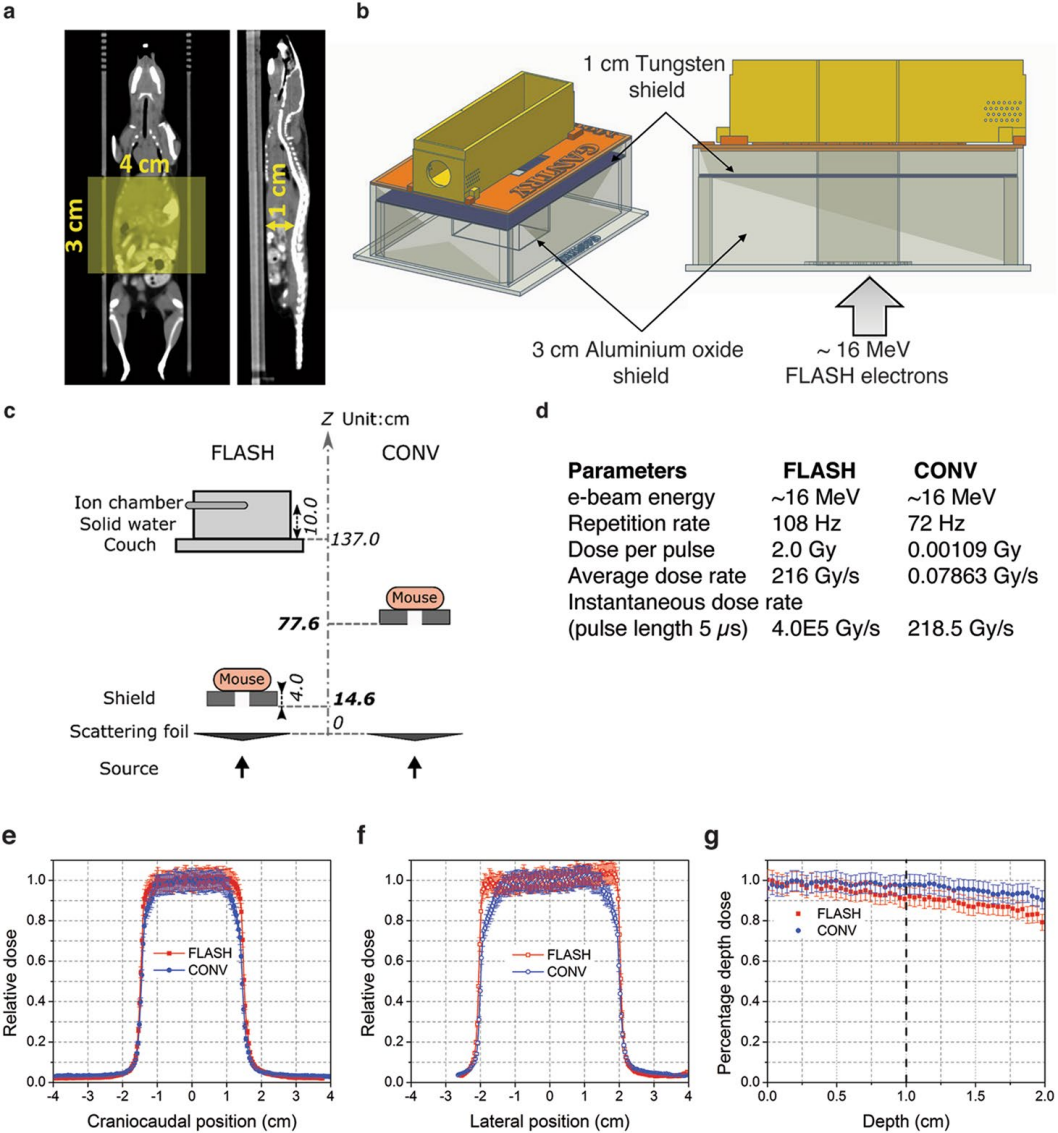
Experimental platform for ultra-high dose rate FLASH and conventional abdominal irradiation. (**a**) CT images (coronal and sagittal slices) showing reproducible mouse positioning within the stereotactic frame. The yellow shaded rectangle (3 cm × 4 cm) indicates the irradiated region in the abdomen; the 3 cm length with the cranial border at the 10^th^ rib encompasses the entire small bowel. The distance from the abdominal wall to the ventral surface of the spine, representing the most posterior extent of the abdominal cavity, is 1 cm (yellow arrow). (**b**) Design of 3-D printed PLA plastic mouse stereotactic positioning frame and abdominal irradiation shield comprising a 3-D printed PLA plastic shell containing layers of 3 cm thick aluminum oxide (Al_2_O_3_) powder in tandem with a mixture of 1 cm thick tungsten beads (2 mm diameter) and powder. The Al_2_O_3_ layer slows down the ~ 16 MeV primary electrons and the tungsten layer stops the transmitted electrons and attenuates the bremsstrahlung x-rays produced by the electron beam. (**c**,**d**) Schematic of the geometric and beam parameters for FLASH and CONV setups. The shorter distance from the electron scattering foil and higher charge per pulse in FLASH produces an average dose rate 2750 times the CONV dose rate. (**e**) Craniocaudal, (**f**) lateral profiles at the entrance surface, and (**g**) depth dose profiles for FLASH and CONV setups were recorded using EBT3 Gafchromic films between layers of polystyrene. The doses are uniformly distributed (within < 10% heterogeneity) within the abdominal region.

**Figure 2:**
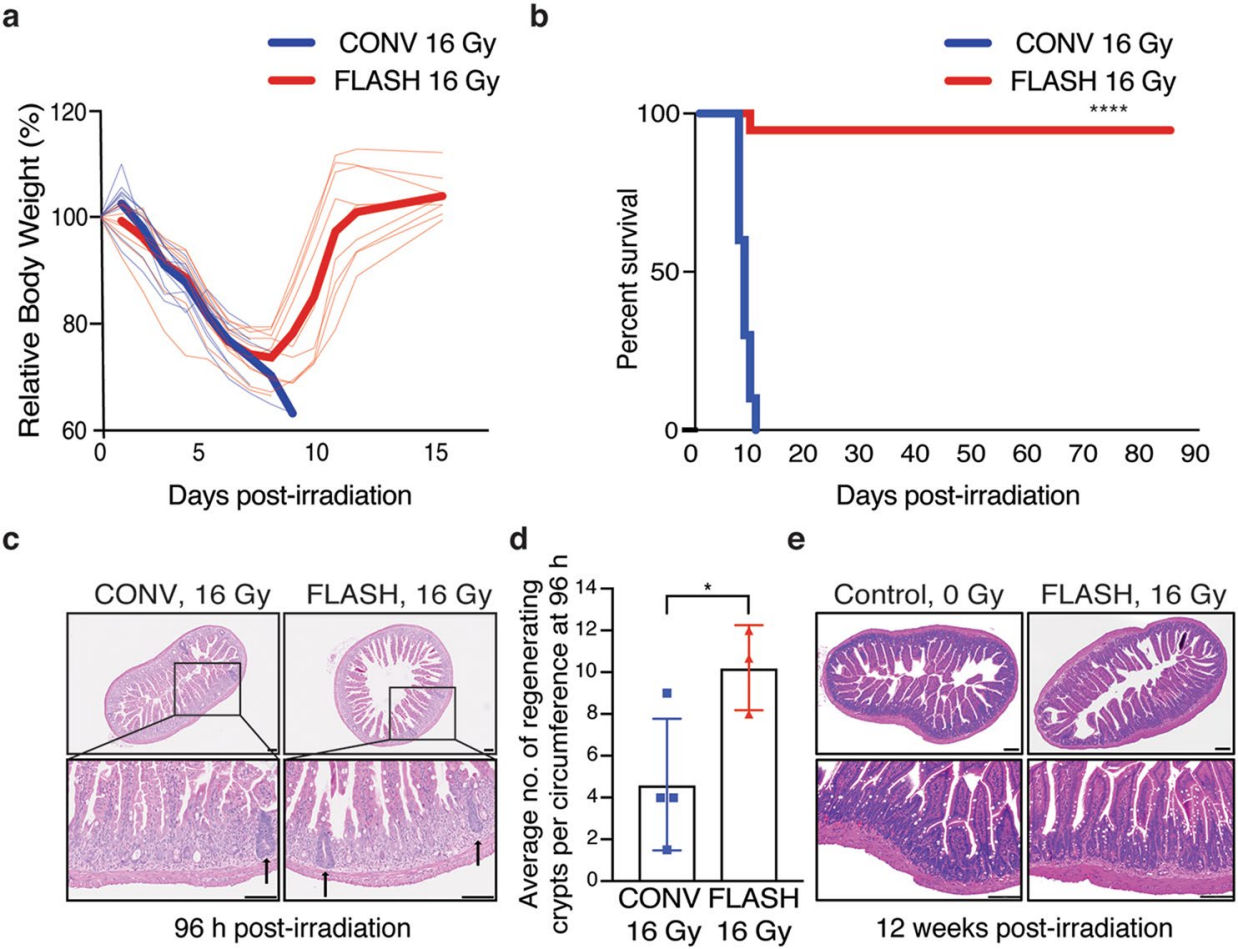
Abdominal FLASH irradiation produces less lethality from radiation-induced gastrointestinal syndrome than CONV irradiation in non-tumor bearing mice. (**a**) Relative percent body weight (%) of each mouse over time after 16 Gy CONV or FLASH TAI; bold lines represent the averages for the cohorts. (**b**) Kaplan–Meier survival curve of animals that received 16 Gy total abdominal irradiation (TAI), demonstrating complete lethality after CONV but near-complete survival after FLASH irradiation (n = 10 per group, **** by log-rank (Mantel-Cox) test p < 0.0001). (**c**) Histological images of hematoxylin and eosin (H&E) stained jejunal sections from animals 96 h after 16 Gy TAI. Arrows point to regenerating crypts. (**d**) Quantification of the average number of regenerating crypts per circumference 96 h post 16 Gy TAI, demonstrating over double the number of regenerating crypts after FLASH vs. CONV irradiation. Regenerating crypts were counted in 3 mid-jejunal circumferences per mouse (n = 4 mice in CONV; n = 3 in FLASH, *p < 0.05 by unpaired 2-tailed Student’s t-test). (**e**) Histological images of H&E stained jejunum cross-sections from unirradiated mice and surviving mice 12 weeks after 16 Gy FLASH TAI, demonstrating normal histology after recovery from FLASH. Scale bar: 100 μm. Error bars represent standard deviation of the mean.

To investigate the safety of abdominal FLASH irradiation, we first examined the effect of abdominal FLASH irradiation in radiation-induced lethal injury. For this purpose, we developed a mouse stereotactic positioning frame with an irradiation field that extends 3 cm in the cranial/caudal direction starting at the 10th rib allowing for abdominal treatment while sparing the pelvic region (Fig. 1a). We next utilized a clinical linear accelerator modified to generate a 16 MeV electron beam to deliver both uniform treatment across the mouse and a homogenous depth dose (within < 10% heterogeneity) throughout the mouse abdominal cavity (1.0 cm maximum depth) in both FLASH (216 Gy/s) and conventional mode (0.079 Gy/s) (Fig. 1b–g). We monitored body weight loss and survival of female C57BL/6 mice irradiated with 16 Gy FLASH or CONV abdominal irradiation. FLASH and CONV irradiated mice lost an average of 26% and 30% of their body weight respectively at day 8 following 16 Gy TAI (Fig. 2a). The majority of FLASH irradiated mice (9/10) recovered to their original body weight and survived more than 90 days post-irradiation (Fig. 2a,b). In contrast, CONV irradiated mice continued to lose body weight and died by day 10 following irradiation (Fig. 2a,b). Complete blood count analysis 96 h post-irradiation demonstrated no significant hematopoietic toxicity after abdominal CONV or FLASH irradiation, indicating that the observed toxicity was not attributable to bone marrow suppression (Supplementary Figure S1a–d). Histologic analysis of the jejunum revealed that while all mice exhibited radiation-induced mucosal damage, FLASH irradiated mice had a twofold increase in the number of regenerating crypts at 96 h post-irradiation compared to CONV irradiated mice (Fig. 2c,d). Notably, the intestinal mucosa of the surviving 16 Gy FLASH irradiated mice was histologically indistinguishable from unirradiated control animals at 12 weeks post irradiation (Fig. 2e). These findings demonstrate that FLASH irradiation produces less radiation-induced lethal injury.

### Abdominal FLASH irradiation spares intestinal function and epithelial integrity compared to CONV irradiation

**Figure 3:**
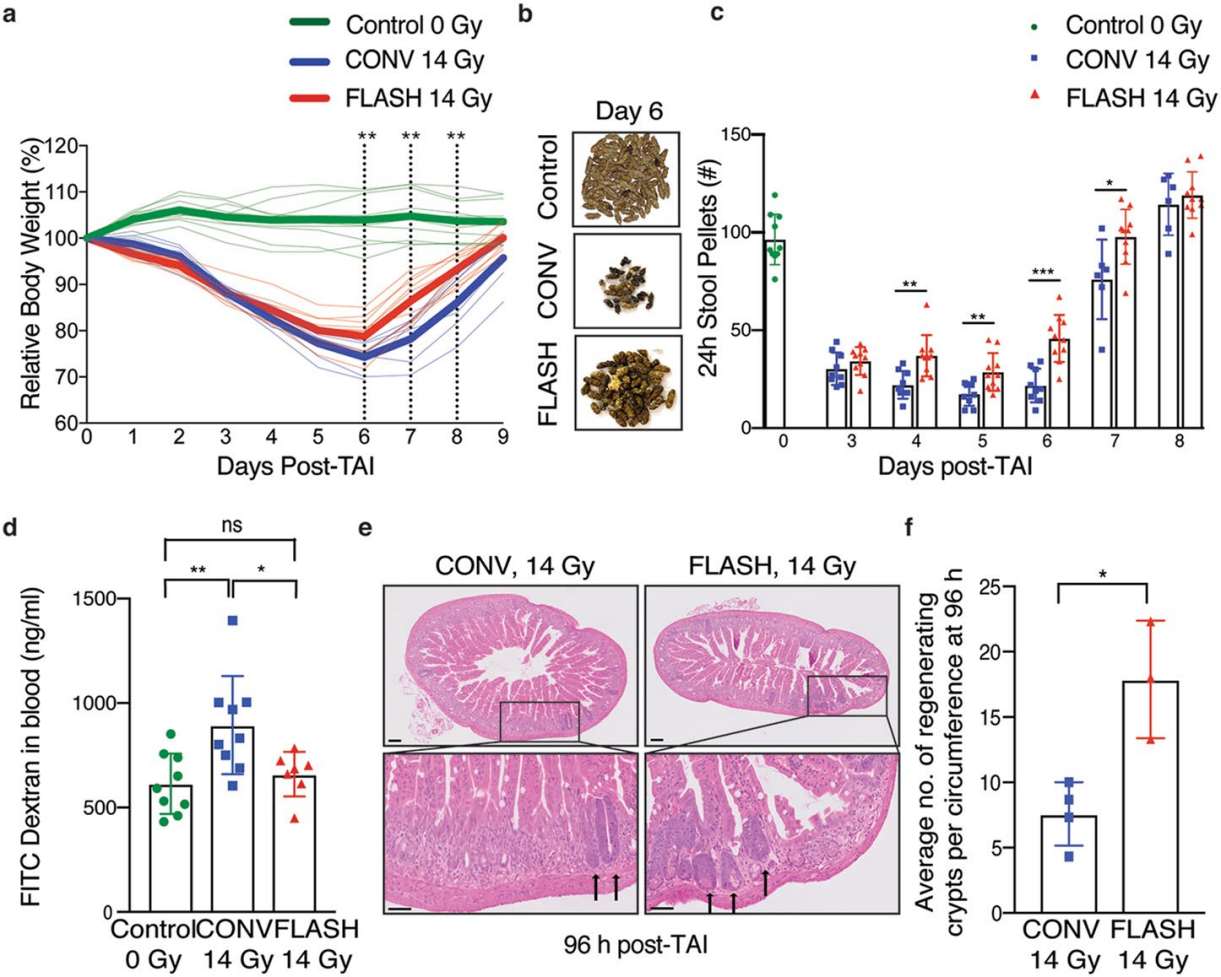
Abdominal FLASH irradiation spares intestinal function and epithelial integrity compared to CONV irradiation at a sub-lethal dose in non-tumor bearing mice. (**a**) Relative body weight (%) of mice over time after 14 Gy CONV or FLASH TAI. Bold lines show the averages for the cohorts. The dotted lines indicate days at which the difference between CONV and FLASH is significant. (n = 10 mice in unirradiated control and FLASH; n = 9 in CONV; n = 6 in CONV on days 7, 8 and 9). (**b**) Representative images of formed stool pellets from singly housed unirradiated control mice and mice six days after sub-lethal 14 Gy TAI, showing an increase in stools after FLASH vs. CONV. (**c**) Quantification of the number of formed stool pellets excreted over 24 h at the indicated time points from unirradiated control mice and mice after 14 Gy TAI, demonstrating less severe decrement and faster recovery after FLASH vs. CONV irradiation (n = 10 mice in unirradiated control & FLASH; n = 9 in CONV; n = 6 in CONV on days 7 and 8). (**d**) Quantification of FITC-dextran in serum at 96 h after 14 Gy TAI, demonstrating loss of intestinal barrier function after CONV but not FLASH irradiation (n = 9 mice in unirradiated control and CONV; n = 7 in FLASH). (**e**) Histological images of H&E stained jejunal sections from animals 96 h after 14 Gy TAI. Arrows point to regenerating crypts. (**f**) Quantification of the average number of regenerating crypts per jejunal circumference 96 h after 14 Gy TAI, demonstrating over double the number of regenerating crypts after FLASH vs. CONV irradiation (n = 4 mice in CONV; n = 3 in FLASH; 3 circumferences per mouse were analyzed). *p < 0.05, **p < 0.01, ***p < 0.001. CONV vs. FLASH compared by unpaired 2-tailed Student’s t-test. Unirradiated control vs. CONV or FLASH compared by one-way ANOVA followed by Tukey’s multiple comparisons post-hoc test. Scale bar shows 100 μm. Error bars represent standard deviation of the mean.

We next evaluated a sublethal dose of irradiation (14 Gy) to compare the effects of FLASH and CONV irradiation on intestinal epithelial integrity and function. Radiation-induced intestinal injury develops when the intestinal epithelium is damaged resulting in fluid and electrolyte loss as well as bacterial translocation. Similar to human patients, mice exposed to high doses of radiation experience abnormal stool production as a result of damage to the intestinal epithelium. By day 3 post-irradiation, both 14 Gy FLASH and CONV irradiated mice exhibited a significant decrease in body weight and the number of formed stool pellets (Fig. 3a,b). However, the FLASH irradiated mice had a faster recovery and smaller decrement of body weight and stool formation compared to CONV irradiated mice (Fig. 3a,b). By day 6, the CONV irradiated mice had 22.6% stool production whereas the FLASH irradiated mice had 47.5% stool production compared to unirradiated controls (Fig. 3b,c). We next compared epithelial integrity following FLASH and CONV irradiation using the FITC-dextran assay where FITC conjugated dextran is fed to the mice and the level of FITC-dextran in the serum reflects the permeability, or loss of barrier function, of the intestinal epithelium^15^. Mice irradiated with 14 Gy abdominal CONV irradiation had an increase in FITC-dextran within the serum at 96 h post-irradiation whereas FLASH irradiated mice had levels comparable to unirradiated control mice (Fig. 3d). Consistent with a functional sparing of the intestine from radiation induced toxicity, we observed a 2.4-fold higher number of regenerating crypts in the jejunum of 14 Gy FLASH irradiated mice compared to 14 Gy CONV irradiated mice (Fig. 3e,f). Notably, an increase in regenerating crypts was also observed in 12 Gy FLASH irradiated mice compared to 12 Gy CONV irradiated mice (Supplementary Figure S2a,b). These findings demonstrate that abdominal FLASH irradiation produces less radiation-induced intestinal injury following abdominal irradiation in healthy mice.

### Abdominal FLASH irradiation alters the proliferation kinetics in crypt cell regeneration compared to CONV irradiation

**Figure 4:**
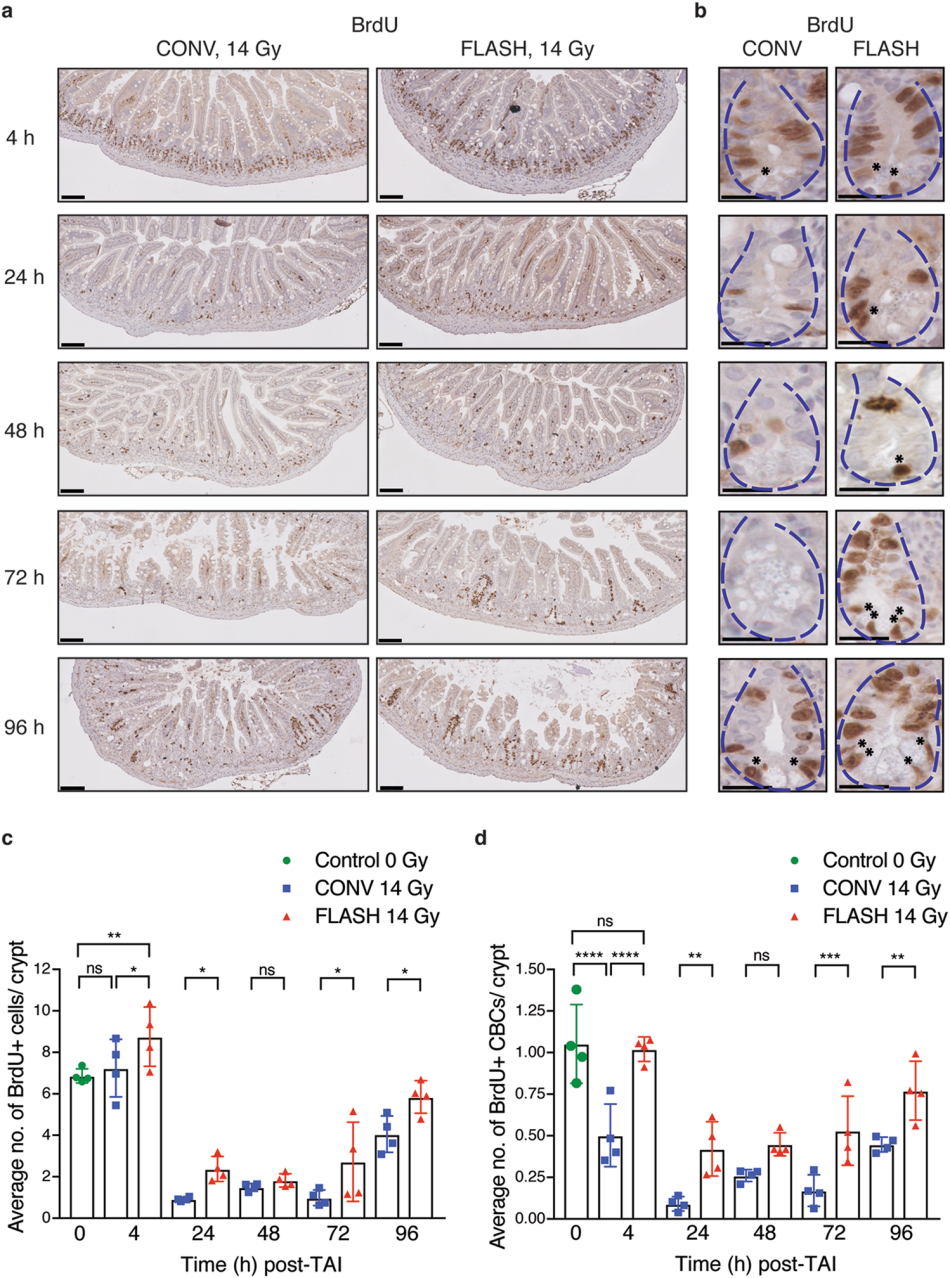
Abdominal FLASH irradiation alters the proliferation kinetics in crypt cell regeneration compared to CONV irradiation in non-tumor bearing mice. Cross sections of small intestines were analyzed for BrdU incorporation and crypt cell proliferation kinetics by BrdU IHC. (**a**) Representative images of BrdU stained jejunum cross sections at 10 × magnification at the indicated time points after sublethal 14 Gy TAI. Scale bar shows 100 μm. (**b**) 40 × magnified crypt images of the jejunum showing BrdU + crypt cells/CBCs at the indicated time points in CONV and FLASH irradiated animals. Scale bar shows 25 μm. Blue dotted lines outline the crypt structure. Black asterisks at the base of the crypt indicate the BrdU + proliferating CBCs in the stem cell zone. The rest of the brown nuclei within the crypt indicate BrdU + cells in the TA zone. (**c**) Quantification of the average number of BrdU + cells per crypt (includes BrdU + cells in both TA zone and stem cell zone). (**d**) BrdU + CBCs per crypt at 4, 24, 48, 72 and 96 h in unirradiated controls and after 14 Gy TAI, demonstrating relative early sparing of proliferating CBCs and more robust regeneration after FLASH *vs*. CONV irradiation. CBC cells were identified based on location at the + 1 to + 3 position wedged between Paneth cells and BrdU + CBCs indicated by black asterisks in the stem cell zone. Fifty crypts per circumference were quantified for BrdU + crypt cells and BrdU + CBCs. Three circumferences per mouse were analyzed (n = 4 mice per group). *p < 0.05, **p < 0.01, ****p < 0.0001; Comparisons by ordinary one-way ANOVA followed by Sidak’s multiple comparisons test. Error bars represent standard deviation of the mean.

To further investigate the effect of FLASH RT on crypt regeneration and to track the crypt proliferation kinetics, we pulse labelled the irradiated animals with BrdU 2 h prior to sacrifice. BrdU pulse labelling captures the response of crypt cells entering S-phase and undergoing DNA replication. It is known that radiation-induced DNA damage rapidly induces the G1/S checkpoint through ATM and p53 dependent mechanisms followed by the emergence of regenerating BrdU + crypts at 96 h post-irradiation^16^. As previously reported, we observed a decrease in the number of BrdU + cells per crypt from 4–72 h post-irradiation, followed by the appearance of BrdU + regenerated crypts at 96 h in the CONV irradiated group (Fig. 4a–c, Supplementary Figure S3a^17^). While the FLASH irradiated mice also had a decrease in BrdU + crypt cells from 4–48 h, BrdU + regenerated crypts began to appear as early as 72 h post-irradiation resulting in a significant increase in BrdU + cells per crypt at both 72 and 96 h timepoints post-irradiation (Fig. 4a–c). These findings further suggest that crypt regeneration is more robust after FLASH compared to CONV irradiation.

Crypt regeneration following radiation injury is mediated by intestinal stem cells located at the base of the crypt. Crypt base columnar (CBC) cells are located at the + 1 to + 3 position wedged between Paneth cells (Supplementary Figure S3b^18^). These cells express the leucine-rich repeat-containing G protein-coupled receptor (Lgr5) and are continuously cycling to maintain intestinal homeostasis^19^. Due to the proliferative nature of Lgr5 + stem cells, they are depleted upon exposure to high dose radiation^20^. Lineage tracing studies have demonstrated that another Bmi + quiescent and radioresistant intestinal stem cell population, located in the + 4 to + 6 region above Paneth cells, compensates for the loss of Lgr5 + cells and gives rise to Lgr5 + cells following injury^20,21^. We therefore sought to investigate whether FLASH alters the proliferation of the CBC stem cell compartment. Interestingly, we found that in comparison to the overall crypt cell population, the number of BrdU + intestinal stem cells in the CBC compartment was reduced by 52% in the CONV irradiated mice at 4 h, whereas the number of BrdU + CBCs in the FLASH irradiated mice were similar to unirradiated control mice (Fig. 4d). By 24 h, the percentage of BrdU + CBCs dropped from 100 to 8% in the CONV irradiated and to 40% in the FLASH irradiated mice (Fig. 4d). By 96 h post-irradiation, 43% and 73% of CBCs were BrdU + in the CONV and FLASH irradiated mice, respectively, correlating with the appearance of regenerating crypts at 96 h post-irradiation (Figs. 3e,f, 4a–d). These data demonstrate that abdominal FLASH irradiation preserves the proliferation of CBCs particularly at early timepoints following irradiation in comparison to CONV irradiation.

### Abdominal FLASH irradiation produces less apoptosis in crypt base columnar cells and modestly less DNA damage than CONV irradiation

**Figure 5:**
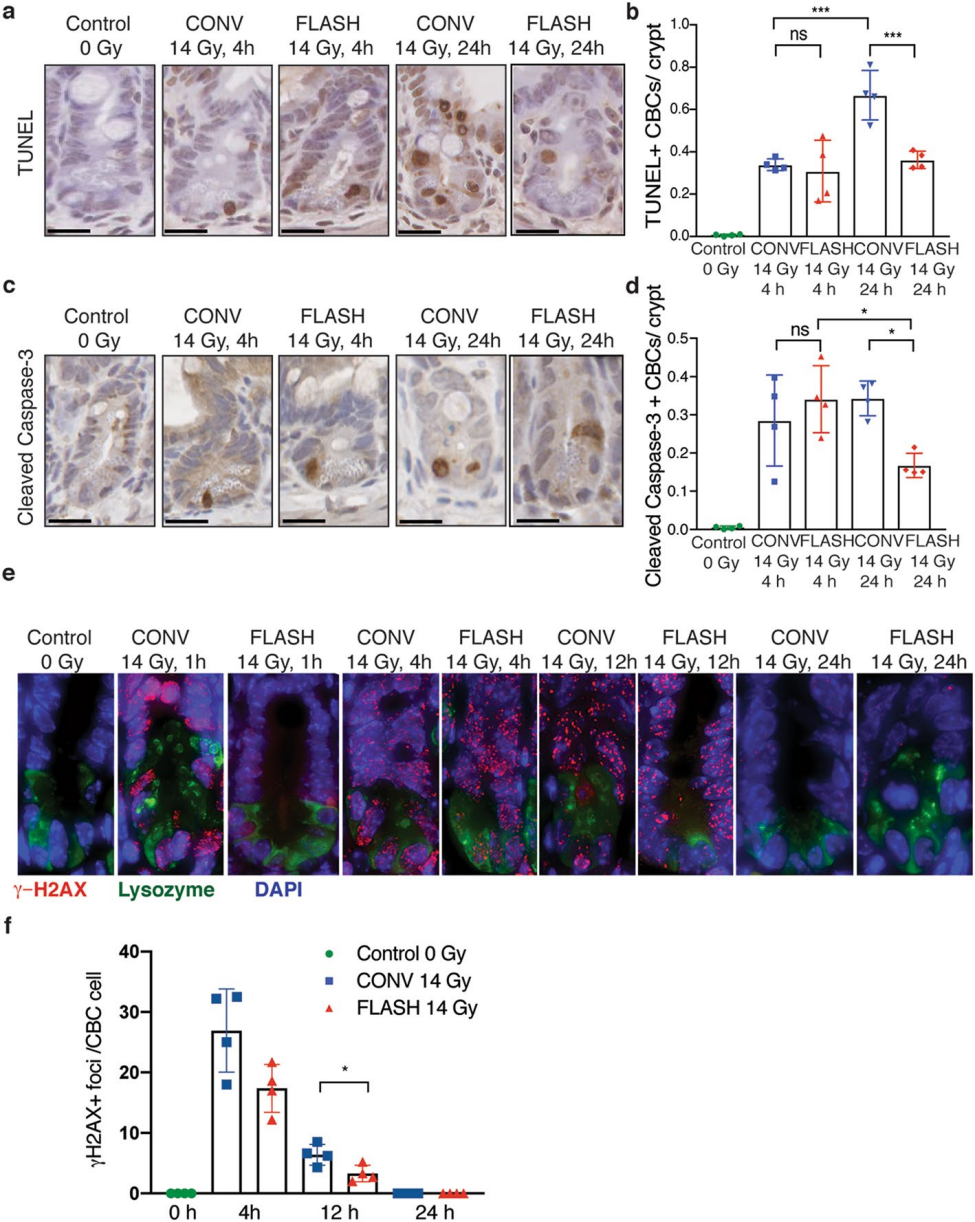
Abdominal FLASH irradiation produces less apoptosis and DNA damage in crypt base columnar cells (CBCs) than CONV irradiation in non-tumor bearing and tumor bearing mice. (**a**) Representative TUNEL stained images of crypts from non-tumor bearing mice at the indicated time points after sham or sublethal 14 Gy abdominal irradiation. (**b**) Quantification of the average number of TUNEL + CBCs per crypt in the jejunum at 4 and 24 h after 14 Gy abdominal irradiation. (**c**) Representative cleaved caspase-3 IHC images of crypts from non-tumor bearing mice at the indicated time points after sham or sublethal 14 Gy abdominal irradiation. (**d**) Quantification of the average number of cleaved caspase-3 + CBCs per crypt in the jejunum at 4 and 24 h after 14 Gy abdominal irradiation. CBC cells were identified based on location at the + 1 to + 3 position wedged between Paneth cells. Apoptosis in CBCs by TUNEL and cleaved caspase-3 was less at 24 h after FLASH *vs*. CONV irradiation. Scale bar shows 25 μm. Crypts in 3 circumferences per mouse were analyzed, n = 4 mice per group. (**e**) Representative γ-H2AX (red), lysozyme (green, a marker of Paneth cells) and DAPI (blue) stained images of crypts from ID8 ovarian tumor bearing mice at the indicated time points after sham or sublethal 14 Gy abdominal irradiation. (**f**) Quantification of the average number of γ-H2AX + foci per CBC cell located in between lysozyme + Paneth cells in the jejunum at 4 h, 12 h and 24 h after 14 Gy abdominal irradiation. n = 4 mice per group. An average of 10 CBC cells per mouse were quantified for γ-H2AX foci. At 4 and 12 h after irradiation, γ-H2AX foci in CBCs were fewer after FLASH than CONV (p < 0.05 at 12 h), and resolved in both groups by 24 h. *ns* no significant difference, *p < 0.05, **p < 0.01, ***p < 0.001; Unirradiated control versus CONV or FLASH compared by one-way ANOVA followed by Tukey’s multiple comparisons test. Error bars represent standard deviation of the mean.

The increase in regenerating crypts in FLASH compared to CONV irradiated mice indicates that intestinal stem cells that repopulate the damaged crypts may be spared from cell death following FLASH irradiation. Therefore, we analyzed crypt cell apoptosis by TUNEL and cleaved caspase-3 staining at 4 and 24 h post-irradiation. Within the entire crypt cell population, there were no significant differences in TUNEL positive or cleaved caspase-3 positive cells between FLASH and CONV irradiation at 4 and 24 h post-irradiation (Supplementary Figure S4a,b). In contrast, the number of TUNEL positive and cleaved caspase-3 positive CBCs, identified based on location at the + 1 to + 3 position wedged between Paneth cells as shown in Supplementary Figure S3b, were decreased in FLASH compared to CONV irradiated mice (Fig. 5a–d). These data indicate that FLASH irradiation produces less apoptosis in intestinal CBCs compared to CONV irradiation.

To begin to investigate the molecular mechanisms by which FLASH irradiation spares radiation-induced cell death, we quantified the number of γ-H2AX positive DNA double strand breaks in the intestinal CBC cells of mice irradiated with 14 Gy FLASH or CONV irradiation over time. One hour after irradiation, we detected extensive γ-H2AX focus formation in CBC cells located in between lysozyme positive Paneth cells of mice treated with abdominal FLASH and CONV irradiation (Fig. 5e). Due to the extensive γ-H2AX foci formation at this early timepoint, individual foci could not be distinguished and quantified accurately. At 4 and 12 h following irradiation, there was a modest 1.5 fold (p = 0.0530) and twofold (p = 0.0309) reduction in the number of γ-H2AX positive foci in CBCs of FLASH irradiated mice compared to CONV irradiated mice respectively (Fig. 5e,f). By 24 h, γ-H2AX positive foci were not detected in CBCs of FLASH or CONV irradiated mice indicating all DNA damage was efficiently repaired by this timepoint (Fig. 5e,f). Together, our findings indicate there is a modest decrease in the initial DNA double strand breaks and/or increase in DNA repair in intestinal CBC cells following abdominal FLASH irradiation.

### Abdominal FLASH irradiation has similar tumor control efficacy as CONV irradiation and spares intestinal function in a preclinical mouse model of ovarian cancer peritoneal metastasis

**Figure 6:**
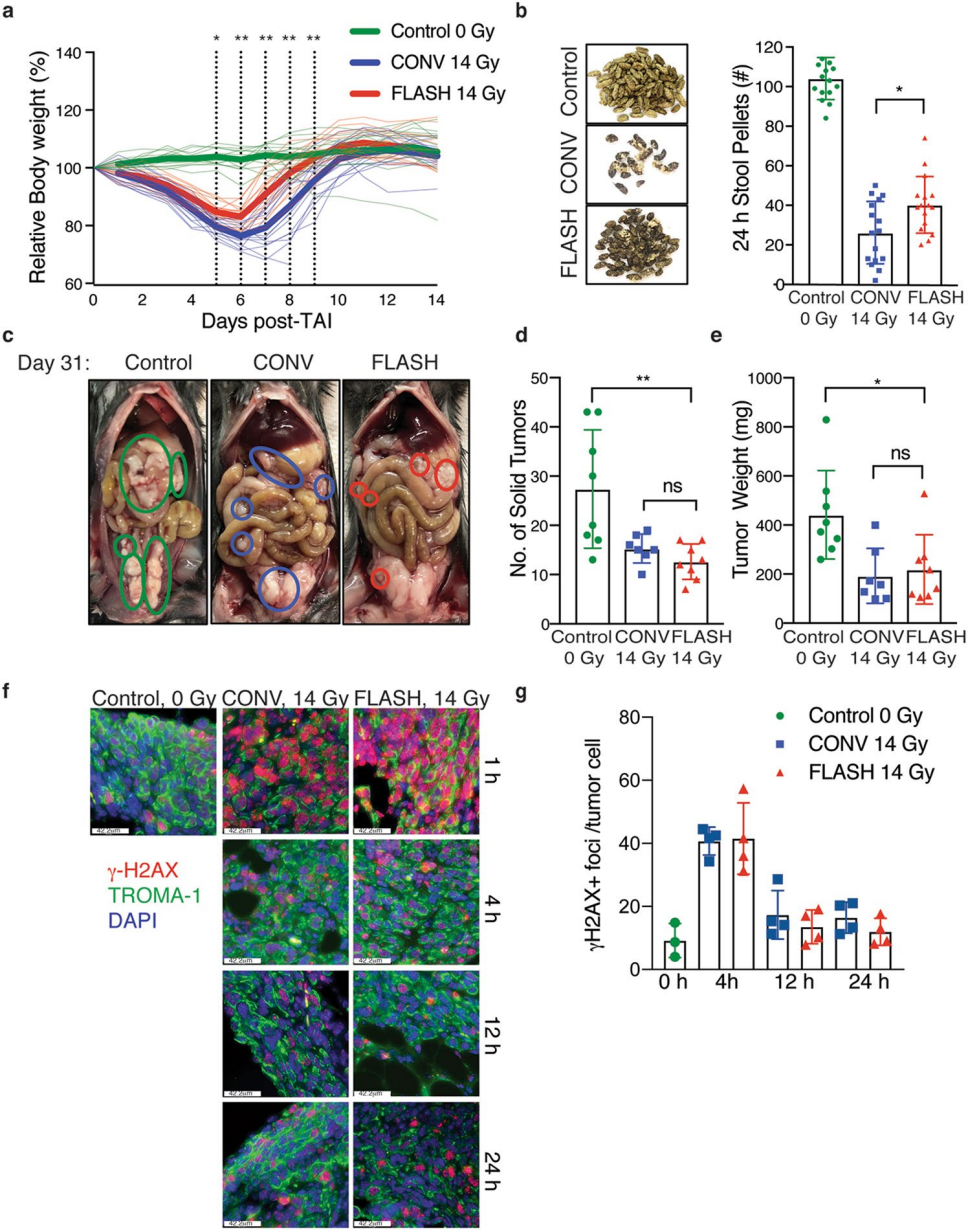
Abdominal FLASH irradiation has similar tumor control efficacy as CONV irradiation and spares intestinal function in a preclinical syngeneic ovarian cancer mouse model. (**a**) Relative body weight (%) of ID8 tumor-bearing mice over time after 14 Gy CONV or FLASH TAI. Bold lines show the average for each cohort. The dotted lines indicate days at which the difference between CONV and FLASH is significant. n = 16 per group. (**b**) Representative images of stool pellets (left) and quantification of the number of stool pellets (right) excreted over 24 h at 15 days after tumor cell injection, or 5 days after 14 Gy abdominal irradiation (n = 16 mice per group), showing increased stools after FLASH *vs*. CONV irradiation, consistent with the observation in non-tumor bearing mice. (**c**) Representative images displaying metastatic tumor burden in mice that were intraperitoneally injected with ID8 ovarian tumor cells and (**d**,**e**) quantification of the number of solid tumor nodules (**d**) and total tumor weights (**e**) in control mice and mice irradiated with 14 Gy CONV or FLASH irradiation, demonstrating similar tumor control efficacy with both FLASH and CONV irradiation. n = 8 mice per group; *ns* no significant difference, *p < 0.05, **p < 0.01; Comparisons by one-way ANOVA followed by Tukey’s multiple comparisons test. Error bars represent standard deviation of the mean. (**f**) Representative γ-H2AX (red), TROMA-1 (green, a marker if ovarian cancer cells) and DAPI (blue) stained images of tumors from ID8 ovarian tumor bearing mice at the indicated time points after sham or sublethal 14 Gy abdominal irradiation. (**g**) Quantification of the average number of γ-H2AX + foci per TROMA-1 positive ID8 cancer cell 4 h, 12 h and 24 h after 14 Gy abdominal irradiation. n = 4 mice per group. An average of 15 TROMA-1 positive cancer cells per mouse were quantified for γ-H2AX foci. At 4 and 12 h after irradiation, γ-H2AX foci in CBCs were similar after FLASH and CONV irradiation and resolved to baseline levels in both groups by 24 h. *ns* no significant difference, *p < 0.05, **p < 0.01, ***p < 0.001; Unirradiated control versus CONV or FLASH compared by one-way ANOVA followed by Tukey’s multiple comparisons test. Error bars represent standard deviation of the mean.

The findings above demonstrate that FLASH irradiation produces less intestinal injury, raising the intriguing possibility that FLASH irradiation may be an effective strategy to enhance the therapeutic index of radiotherapy for abdominal and pelvic tumors, such as ovarian cancer. Therefore, we compared the safety and efficacy of FLASH and CONV RT in a preclinical model of ovarian cancer metastasis. We chose to study the ID8 ovarian cancer peritoneal metastasis model in C57BL/6 mice as the disease metastasizes throughout the peritoneal cavity and forms tumor nodules along the small and large intestine^22^. Moreover, radiation-induced bowel toxicity limits the use of radiation therapy in the treatment of ovarian cancer^23^. In this preclinical study, C57BL/6 mice were injected i.p. with ID8 ovarian cancer cells. At day 10 after injection, the mice were randomized into unirradiated control, 14 Gy CONV, or 14 Gy FLASH irradiation treatment groups. Ninety-six hours post-irradiation, mice were separated and singly housed to quantify stool production. At day 31, when the unirradiated control mice display signs of morbidity, all mice were euthanized, and tumor burden was quantified. FLASH irradiated mice lost an average of 20% of their body weight while CONV irradiated mice lost an average of 25% of their body weight at 6 days after 14 Gy TAI, all of which restored their pre-irradiation bodyweights within 2 weeks after irradiation (Fig. 6a). Moreover, the number of stool pellets excreted over 24 h at day 5 post-irradiation were higher in FLASH compared to CONV irradiated mice (Fig. 6b). These data indicate that FLASH irradiation increases intestinal function in ovarian tumor-bearing mice compared to CONV irradiation. Analysis of total tumor burden revealed a decrease in both the number of tumor nodules and total tumor weights in mice irradiated with both14 Gy TAI CONV and FLASH irradiation when compared to the unirradiated control mice (Fig. 6c–e). No significant differences in tumor number or weights were observed when comparing FLASH to CONV irradiated mice, indicating that FLASH and CONV irradiation have similar efficacy in the treatment of ovarian cancer peritoneal tumors (Fig. 6d,e). Consistent with equivalent tumor cytotoxicity, we observed similar γ-H2AX positive foci in TROMA-1 positive ID8 cancer cells in the omentum of mice at 4, 12 and 24 h following FLASH and CONV irradiation (Fig. 6f,g). Due to extensive γ-H2AX foci formation at the 1 h timepoint following irradiation, individual foci could not be distinguished and quantified accurately. Our results demonstrate that while both abdominal FLASH and CONV irradiation reduce ovarian cancer tumor burden in the peritoneal cavity, FLASH irradiation increased intestinal function in this tumor model.

## Discussion

Ovarian cancer is the 5th leading cause of cancer related deaths among women in the United States. The majority of women diagnosed with ovarian cancer present with advanced stage disease and widespread metastasis throughout the abdomen and pelvis. First-line therapies for patients with ovarian cancer include optimal surgical debulking and platinum-based chemotherapy. While the majority of patients initially respond to platinum-based chemotherapy, most develop recurrent disease and succumb to their disease. With the development of chemoresistance in the majority of patients, the identification of effective therapeutic strategies for the treatment of ovarian cancer is needed.

Our findings have important clinical implications for the treatment of metastatic ovarian cancer. We demonstrate that both FLASH and CONV irradiation have similar tumor control efficacy in a preclinical model of ovarian cancer metastasis. Simultaneously, we demonstrate that abdominal FLASH irradiation spares mice from lethal intestinal injury relative to CONV irradiation. Moreover, at sublethal doses, FLASH irradiation preserves intestinal function and recovery compared to CONV irradiation in both healthy and tumor-bearing mice. These observations of marked radiobiological advantages of FLASH over CONV irradiation in the highly radiosensitive intestine suggest the translational potential of FLASH RT for increased therapeutic index in the treatment of metastatic ovarian cancer. Recently, a preclinical study using a proton beam demonstrated both intestinal sparing and similar control of subcutaneous tumor models by FLASH compared to standard dose rate proton therapy, consistent with our observations using a high-energy electron beam, suggesting that this effect is general across radiation types^24^. Both the FLASH proton beam in that study and the FLASH electron beam in the present study were produced by modifications of clinically available treatment systems. However, a challenge to clinical translation is the limited target volume (or depth in the case of electrons in this energy range) that can be treated at FLASH dose rates using these systems. While the preclinical technologies that have been used for FLASH irradiation of mice do not scale to larger volume, deep-seated tumors in human patients, new radiation therapy technologies to translate FLASH RT to humans are actively under development^25^.

Additionally, studies to determine whether FLASH mediated protection of the intestine from radiation-induced toxicity is maintained in fractionated protocols will also be needed to inform the design of clinical trials. Among the hypotheses of mechanisms underlying increased therapeutic index of FLASH compared to CONV RT are radiochemical depletion of oxygen by FLASH in normal tissue stem cell compartments, and irradiation of a smaller proportion of circulating immune cells during the briefer duration of FLASH RT^26^. The smaller dose per fraction used in fractionated regimens might be insufficient to reach the threshold required to produce radiobiologically significant oxygen depletion, and administering a greater number of fractions might expose a higher cumulative proportion of circulating immune cells to doses sufficient to impair immune function. As such, there is a potential concern that the beneficial FLASH effect may be attenuated in the fractionated regimens used clinically. While we currently lack experimental data to substantiate this concern, these are testable hypotheses.

On the other hand, treatment regimens more directly analogous to the ones we tested in this preclinical study, i.e., large single fraction abdominal irradiation may have translational value if the FLASH effect holds in human patients. We demonstrated that at a dose of abdominal irradiation that was lethal with CONV irradiation, recovery at the histological level was complete after FLASH irradiation. Even if the dose is insufficient to cure metastatic ovarian cancer on its own, it may provide sufficient tumor cytoreduction to augment the effects of systemic therapies including immunotherapies. Furthermore, given the recovery after treatment, it may be possible to administer repeat treatments of high-dose abdominal FLASH RT. These are hypotheses that can be validated experimentally.

The mechanisms of FLASH sparing of the intestine likely stem from the enhanced regenerative potential of intestinal stem cells. Radiation causes damage to the intestinal epithelium by inducing cell death in Lgr5 + intestinal crypt stem cells that regenerate the intestinal epithelial cells^18^. We observed that at a large single dose of 14 Gy, FLASH RT produced modestly fewer γ-H2AX foci and less apoptosis in CBC stem cells, a greater number of regenerating crypts, and preservation of intestinal function and survival. These findings suggest that FLASH irradiation produces less DNA damage and/or alters the DNA damage response in intestinal stem cells to enhance crypt regeneration. Our findings set the stage for future studies investigating the cellular and molecular mechanisms driving the FLASH response in the intestinal stem cell compartment.

Previous studies have suggested that the induced transient hypoxia and its accompanying increase in radioresistance at extremely high dose rates may underlie the FLASH effect in vitro and in vivo^27–29^. Based on radiochemical modeling studies, a 10 Gy or greater dose of FLASH RT has the potential to deplete molecular oxygen in tissues at physiologic oxygen tensions, as oxygen rapidly reacts with radicals formed by radiolysis of water and other biomolecules (ROS)^30^. Perhaps especially in stem cell niches that may have baseline hypoxia, transient anoxia produced by FLASH but not CONV irradiation would result in reduced DNA double strand breaks caused by reduced oxygen-mediated fixation of DNA damage^26,31–33^. In support of this hypothesis, Montay-Gruel et al*.* found that carbogen breathing to increase tissue oxygenation partially abrogated the cognitive sparing observed after FLASH brain irradiation^34^. We demonstrate that FLASH RT may produce less early DNA damage within intestinal crypt cells in vivo compared to CONV RT, which would be consistent with the oxygen depletion hypothesis. Future studies to measure tissue oxygen and ROS levels in real time during FLASH and CONV irradiation, which may be enabled by oxygen and ROS sensing nanoparticle probes, would provide the most direct evidence of this mechanism^35,36^.

In summary, our findings suggest that with continued development, FLASH irradiation may offer an opportunity to reintroduce radiotherapy into the armamentarium of ovarian cancer therapeutics. Historical data demonstrate that the majority of ovarian tumors respond to radiotherapy. Therefore, FLASH irradiation has the potential to extend overall survival and improve quality of life for women diagnosed with advanced epithelial ovarian cancer.

## Methods

### Study design

The goal of this study was to investigate the safety and efficacy of total abdominal FLASH RT (TAI-FLASH) compared to conventional dose rate RT (TAI-CONV) in mice. All procedures for use of animals and their care were approved by the Institutional Animal Care and Use Committee of Stanford University in accordance with institutional and NIH guidelines. Six to eight-week-old female C57BL/6 mice (Jackson Labs) were irradiated by both CONV and FLASH methods. Mice were anaesthetized with a mixture of ketamine (100 mg/kg) and xylazine (10 mg/kg) injected into the peritoneum. Control mice were treated with the same dose of ketamine/xylazine but were not exposed to radiation. Safety of TAI-FLASH relative to TAI-CONV was first assessed in non-tumor bearing mice. At a lethal dose of 16 Gy (when delivered by CONV), mice were irradiated by FLASH and CONV methods and the overall survival and the number of regenerating crypts within the jejunum were determined. The effects of FLASH and CONV irradiation on gastrointestinal function and epithelial integrity were evaluated at a sublethal dose of 14 Gy. GI function and integrity were measured by the number of stool pellets excreted over 24 h, regenerating crypts and FITC dextran assay 96 h post-irradiation. Mechanisms behind the normal tissue-sparing effect of FLASH irradiation was investigated by studying crypt regeneration, cell proliferation kinetics, apoptotic cell death and γ-H2AX foci formation at 0, 4, 12, 24, 48, 72 and 96 h after irradiation. The efficacy of abdominal FLASH RT and CONV RT was then compared in a preclinical model of ovarian cancer metastasis. ID8 ovarian cancer peritoneal metastasis was established in C57BL/6 mice. Analysis of macroscopic tumor burden and tumor weight as well as GI function as described above was performed to demonstrate tumor control efficacy while sparing normal GI function.

### Mouse irradiation

We developed a custom mouse stereotactic positioning frame made of PLA plastic using 3-D printing. Reproducible positioning within the frame was achieved by registering the front teeth on a nylon filament at a fixed location at the cranial end of the frame with extension of the hindlimbs and tail through designated slots at the caudal end of the frame (Fig. 1b). Positioning reproducibility to within 1 mm was confirmed by microCT imaging in a representative cohort of mice (Fig. 1a). An abdominal irradiation shield was made by 3-D printing a PLA plastic shell with a central opening of 4 cm (lateral) by 3 cm (craniocaudal). Internally, a 3 cm thick layer of aluminum oxide powder in tandem with a 1 cm thick layer of tungsten spheres (2 mm diameter) was placed, a combination designed to minimize leakage dose from bremsstrahlung radiation produced in the shield materials (Fig. 1b). The stereotactic frame was registered to the shield such that the opening extended from the tenth rib at the cranial border to 3 cm caudally, consistently encompassing all of the small intestine. EBT3 Gafchromic film (Ashland Advanced Materials, Bridgewater NJ) dosimetry confirmed that leakage dose to the shielded portions of the body when irradiating with 16 MeV electrons was < 3.5% of the central dose at 10 mm from the field edge and further.

The shield and positioning frame were loaded into a polystyrene cradle registered to specified locations relative to the LINAC treatment head for FLASH and CONV irradiation. For both FLASH and CONV setups, we placed EBT3 Gafchromic films between layers of polystyrene to measure transverse and depth dose profiles to characterize dose homogeneity throughout the treatment volume (Fig. 1e–g). In addition, entrance dose for every individual mouse irradiation was recorded by EBT3 Gafchromic films (1 × 2 inch) placed inside the positioning frame. For both FLASH and CONV irradiation, the dose was prescribed at the entrance surface of the mouse.

### CONV irradiation setup

We used a Varian Trilogy radiotherapy system (Varian Medical Systems, Palo Alto, CA) to perform both CONV and FLASH irradiation. For CONV irradiation, the gantry was rotated to 180 degree (beam direction from floor to ceiling) and the collimator was rotated to 0 degree. The cradle with the shield and mouse jig was placed on top of a 15 cm electron applicator, supported by a 1 cm thick lead sheet with a 3.5 × 5 cm opening that also served as a primary shield, such that the distance from the electron scattering foil to the shield was 77.6 cm. Under service mode, irradiation was delivered using a clinical 16 MeV electron beam in the 400 MU/min dose rate mode (pulse repetition rate 72 Hz). Calibration by film dosimetry determined that the entrance dose after the shield was 1.18 cGy/MU, with a resulting average dose rate of 0.079 Gy/s (dose per pulse of 0.00109 Gy).

### FLASH irradiation setup

We configured the Varian Trilogy radiotherapy system to perform FLASH irradiation as previously described^37^. The gantry was rotated to 180 degree, the treatment head cover was removed, and the jaws were fully opened (40 × 40 cm). The cradle with radiation shield and mouse stereotactic frame was loaded and registered to fixed points on the face of the gantry, such that the distance from the electron scattering foil to the shield was 14.6 cm. Beam parameters were configured on a dedicated electron beam control board. We used an electron beam energy of approximately 16 MeV with the 16 meV scattering foil (confirmed by depth dose measurements) and adjusted the radiofrequency power and gun current settings to produce a dose per pulse of 2.0 Gy at the entrance surface of the mouse. We controlled pulse delivery using a programmable controller board (STEMlab 125-14, Red Pitaya, Solkan, Slovenia) and relay circuit to count the number of delivered pulses detected by the internal monitor chamber and impose beam hold and release through the respiratory gating system of the LINAC. We used an external ion chamber positioned after the mouse and 10 cm of solid water (where the dose rate did not saturate the chamber), calibrated to film measurements of entrance dose, to provide immediate dose readout per mouse. The pulse repetition rate was set to 108 Hz for an average dose rate of 216 Gy/s at 2 Gy/pulse at the entrance surface of the mouse.

### Ovarian cancer tumor model

The mouse ID8 ovarian cancer cell line was obtained from Dr. Katherine F. Roby, University of Kansas Medical Center, Kansas City, KS^22^. The cell line was authenticated from the original source and was used within 6 months of receipt. Additionally, cells were tested upon receipt for viability, cell morphology and the presence of *Mycoplasma* and viruses (Charles River Laboratories). ID8 cells were passaged in DMEM supplemented with 10% FCS and Pen/Strep. Cells (5 × 10^6^ cells in 200 μl PBS) were administered via intraperitoneal injection using a 27-gauge needle (BD). Injected animals were irradiated with FLASH or CONV RT on day 10 post-inoculation. Animals were euthanized on day 31 and tumor burden was assessed.

### Survival and intestinal function analysis

Survival times of mice were measured from irradiation until death. Criteria for early euthanasia specified in our APLAC protocol are based on the Mouse Intervention Scoring System (MISS 3) score of 12 or greater as previously described^38^. The MISS 3 scoring system takes into consideration the appearance, respiratory rate, general behavior, provoked behavior, and weight loss. No mice in our study survived with an MISS3 score of 12.

Intestinal function was assessed by measuring daily weights and by counting the number of formed stool pellets at specified time points, as diarrhea and inability to make stools is an indicator of radiation-induced gastrointestinal syndrome. Animals were singly housed for 24 h prior to stool collection, after which their droppings were separated from the cage bedding and counted. Animals were then returned to their original cage groupings.

Intestinal barrier function was assessed in a cohort of mice by the FITC-dextran assay. FITC-dextran (Sigma-Aldrich) was prepared as a stock solution at a concentration of 100 mg/mL in PBS. FITC-dextran was administered via oral gavage at a dose of 0.6 mg FITC-dextran/g of body weight at 96 h post-irradiation. Mice were sacrificed after 4 h and blood was collected by cardiac puncture. After incubating 2 h at room temperature, serum was isolated by centrifugation at 2000×*g* for 20 min at 4 °C. The amount of FITC-dextran in the serum was determined by measuring the fluorescence at an excitation wavelength of 485 nm and an emission wavelength of 525 nm using a plate reader (Synergy microplate reader, H1 Biotek).

### Tissue processing and histological analysis

Animals were euthanized via CO2 asphyxiation and secondary cardiac exsanguination. Soft tissues were harvested and immersion-fixed in 10% neutral buffered formalin for 24 h, followed by PBS for 24 h and then stored in 70% ethanol. Three transverse sections of the small intestine from the jejunum (mid-segment) were collected. Formalin-fixed tissues were processed routinely, embedded in paraffin, sectioned at 5 μm and stained with hematoxylin and eosin.

### Regenerating crypt counts

A total of three transverse sections of jejunum were analyzed per mouse for the number of regenerating crypts by the crypt microcolony assay^39^. Transverse sections were analyzed if they met the following criteria: (1) a complete jejunal circumference was present; and (2) the mucosa was oriented perpendicular to the long axis of the intestine. Crypts were considered regenerating if they comprised > 10 basophilic crypt epithelial cells (n = 4 mice/ group).

### γ-H2AX immunofluorescence

Slides were baked and deparaffinized in xylene and alcohol series followed by heat-mediated antigen retrieval using sodium citrate buffer. Tissue sections were then blocked with the Mouse On Mouse (M.O.M.) blocking reagent for 1 h (Vector Labs) and serum-free protein blocking agent (Dako) and then incubated with mouse mAb for Phospho-Histone H2A.X (Ser139, 9F3, Abcam) with rabbit anti-Lysozyme (EC 3.2.1.17, DAKO) for jejunum sections or rat anti-TROMA1 (DSHB) for ovarian tumor sections at 4 °C overnight. Slides were washed in 1X PBS and incubated with Alexafluor 594 conjugated anti-mouse secondary antibody, Alexaflour 488 anti-rabbit or Alexaflour 488 anti-rat for 30 min at 37 °C. 1X PBS containing 0.1% BSA and 0.2% Triton X-100 were used to dilute the antibodies. The slides were then DAPI stained. Images were captured as Z-stacks using a fluorescence microscope (Leica DMi8). Thirty one slices with a step size of 0.4 μm (z-direction) between slices were captured/z-stack to map the entire nucleus^40^. At least three 60× images were collected randomly for each time point. Maximum intensity projections of the captured Z-stacks were analyzed for γ-H2AX foci. An average of 10 CBC cells, located in between lysozyme positive Paneth cells per mouse were analyzed for the number of punctate γ-H2AX foci in jejunal circumferences using the Fiji ImageJ software (n = 4 mice/ group).

### TUNEL assay

TUNEL assay was performed using ApopTag Peroxidase In Situ Apoptosis Detection Kit (Millipore) according to manufacturer’s instructions. Briefly, deparaffinized sections were enzymatically digested using 20 μg/ml Proteinase K and quenched in hydrogen peroxide before treating with TdT enzyme and anti-digoxigenin peroxidase conjugate. Signals were developed using DAB peroxidase substrate and signal development was monitored under a light microscope. Slides were scanned using Nanozoomer (Hamamatsu). Number of TUNEL + crypt cells and TUNEL + crypt base columnar cells (CBCs) were quantified per crypt per circumference. Cells were considered positive if they exhibited dark brown nuclear staining. Three jejunal circumferences per mouse were analyzed (n = 4 mice/ group).

### Cleaved caspase-3 immunohistochemistry

Antigen retrieval was performed in 10 mM sodium citrate buffer. Following avidin/biotin block and serum-free protein block, sections were incubated in cleaved caspase-3 (Asp175) antibody (1:300; Cell Signaling Technology) at 4 °C overnight in a humidified chamber. Biotinylated secondary antibody, ABC kit and DAB substrate were employed to develop the signal. Slides were scanned using Nanozoomer. Number of cleaved caspase-3 + crypt cells and cleaved caspase-3 + CBCs were quantified per crypt per circumference. Three jejunal circumferences per mouse were analyzed (n = 4 mice/group).

### BrdU immunohistochemistry

BrdU (Sigma) was intraperitoneally injected two hours prior to sacrificing the animals at a dose of 100 mg/kg in sterile PBS. Tissues were serially harvested at their respective timepoints following irradiation, fixed, paraffin-embedded and sectioned. Paraffin sections (5 μm) were baked, deparaffinized and subjected to heat-mediated antigen retrieval in Tris–EDTA pH 9.0. Antigen retrieval was performed at high pressure for 5 min in a pressure cooker. Slides were cooled on ice, rinsed in water and quenched for endogenous peroxidase in 0.3% H_2_O_2_ at room temperature. Sections were permeabilized in 1X TBS containing 0.025% Triton X-100 (TBS-TX) for 10 min, blocked with TBS-TX containing 10% goat serum and 1% BSA for 30 min at room temperature. Tissue sections were incubated with BrdU mouse monoclonal biotinylated primary antibody (1:100; Invitrogen) at 4 °C overnight followed by biotinylated anti-mouse secondary antibody. Signals were amplified using Vecta Elite ABC kit and monitored under a light microscope using DAB peroxidase substrate kit. Sections were counter-stained with hematoxylin, dehydrated, and mounted. Slides were scanned using NanoZoomer. Fifty crypts per circumference were quantified for BrdU + crypt cells and BrdU + CBCs. Three jejunal circumferences per mouse were analyzed (n = 4 mice/group).

### Complete blood count analysis

Blood was collected by cardiac puncture at the time of sacrifice. Complete blood counts data were collected by analyzing the blood using a Hemavet 950FS (Drew Scientific).

### Statistical analysis

Analysis of two groups with a continuous outcome were performed using a Student’s t-test. Analyses of three groups or more with a continuous outcome were performed using an ANOVA and pair-wise comparisons were performed in a post hoc analysis with a Tukey or Sidak adjustment for multiple comparisons. Error bars represent standard deviations. Time to event outcomes were summarized with Kaplan–Meier curves and groups were compared in a log-rank test. Analyses of continuous outcomes measured at multiple time points were performed using a mixed effects model with pair-wise comparisons done in a post hoc test with a Tukey adjustment for multiple comparisons. All statistical tests were two-sided with an alpha level of 0.05. All analyses were performed in SAS v 9.4 (SAS Institute Inc., Cary, NC) or Prism v 8.3.0 (GraphPad Software, San Diego, CA).

## Supplementary information

Supplementary Information
