# Supplementary material for: Abdominal FLASH irradiation reduces radiation-induced gastrointestinal toxicity for the treatment of ovarian cancer in mice
Source: Sci Rep. 2020 Dec 10;10:21600. doi: 10.1038/s41598-020-78017-7 (PMC7728763; doi:10.1038/s41598-020-78017-7)
Supplement: Supplementary file 1 — Supplementary Information [file 41598_2020_78017_MOESM1_ESM.pdf]

Abdominal FLASH irradiation reduces radiation-induced gastrointestinal toxicity for the treatment of ovarian cancer in mice

Karen Levy[1,2]*, Suchitra Natarajan[1]*, Jinghui Wang[1]*, Stephanie Chow[1,2], Joshua T. Eggold[1], Phoebe Loo[1], Rakesh Manjappa[1], Stavros Melemenidis[1], Frederick M. Lartey[1], Emil Schüler[1], Lawrie Skinner[1], Marjan Rafat[1,5], Ryan Ko[1], Anna Kim[1], Duaa Al Rawi[1], Rie von Eyben[1], Oliver Dorigo[2], Kerriann M. Casey[3], Edward E. Graves[1,4], Karl Bush[1], Amy S. Yu[1], Albert C. Koong[6], Peter G. Maxim[7], Billy W. Loo Jr.[1,4]**, Erinn B. Rankin[1,2,4]**

[1]Department of Radiation Oncology, Stanford University School of Medicine, Stanford CA 94305.
[2]Department of Obstetrics and Gynecology, Stanford University School of Medicine, Stanford CA 94305.
[3]Department of Comparative Medicine, Stanford University School of Medicine, Stanford CA 94305.
[4]Stanford Cancer Institute, Stanford University School of Medicine, Stanford CA 94305.
[5]Department of Chemical and Biomolecular Engineering, Vanderbilt University School of Engineering, Nashville TN 37235.
[6]Division of Radiation Oncology, University of Texas MD Anderson Cancer Center, Houston TX 77030.
[7]Department of Radiation Oncology, Indiana University School of Medicine, Indianapolis IN 46202.

*, ** These authors contributed equally to the work.

Running Title: FLASH irradiation reduces radiation-induced gastrointestinal injury.

Keywords: Ovarian cancer, radiation therapy, FLASH, intestinal injury, crypt base columnar (CBC) cells

**Correspondence should be addressed to:

Billy W. Loo Jr. (bwloo@stanford.edu)
Department of Radiation Oncology and Stanford Cancer Institute
Stanford University School of Medicine
875 Blake Wilbur Drive
Stanford, CA 94305

Erinn B. Rankin (erankin@stanford.edu) – Contact for editorial correspondence
Departments of Radiation Oncology and Obstetrics and Gynecology, and Stanford Cancer Institute
Stanford University School of Medicine
269 Campus Drive
1245 CCSR
Stanford, CA 94305

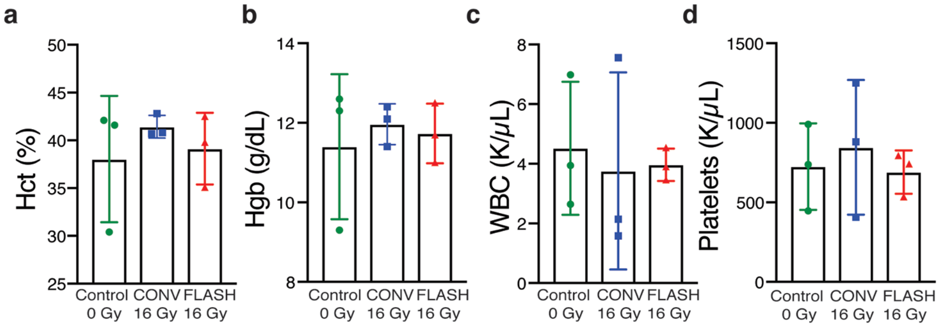

Supplementary Figure 1: Abdominal 16 Gy FLASH and CONV irradiation does not induce hematologic toxicity in mice.

(a-d) Circulating blood cell counts (hematocrit (a), hemoglobin (b), WBCs (c), and platelets (d)) in unirradiated control mice and irradiated mice 96 h after 16 Gy TAI show no significant hematologic toxicity from either FLASH or CONV abdominal irradiation. Blood was drawn by cardiac puncture at the time of euthanasia. ns=no significant difference by one-way ANOVA and Tukey's multiple comparisons test. Error bars represent standard deviation of the mean.

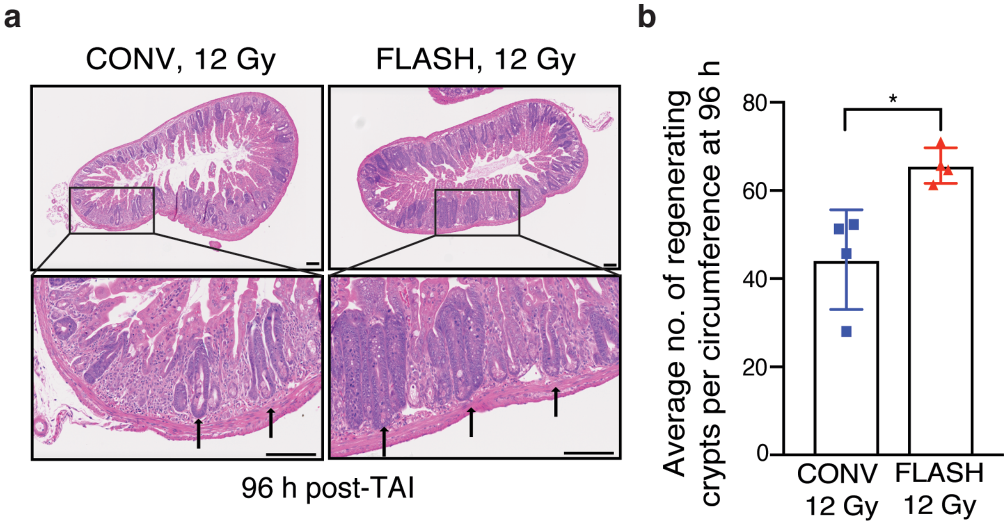

Supplementary Figure 2: Abdominal FLASH irradiation spares intestinal function and epithelial integrity compared to CONV irradiation at a sub-lethal dose in non-tumor bearing mice.

(a) Histological images of hematoxylin and eosin (H&E) stained jejunal sections from animals 96 hours (h) after 12 Gy TAI. Scale bar show 100 µm. Arrows point to regenerating crypts. **(b)** Quantification of the average number of regenerating crypts per circumference 96 hours (h) after 12 Gy TAI, demonstrating a higher number of regenerating crypts after FLASH *vs*. CONV irradiation. Regenerating crypts were counted in 3 circumferences per mouse. n=4 mice per group. $^*p<0.05$, $^{**}p<0.01$, $^{***}p<0.001$. CONV *vs*. FLASH compared by unpaired 2-tailed Student's t-test. Error bars represent standard deviation of the mean.

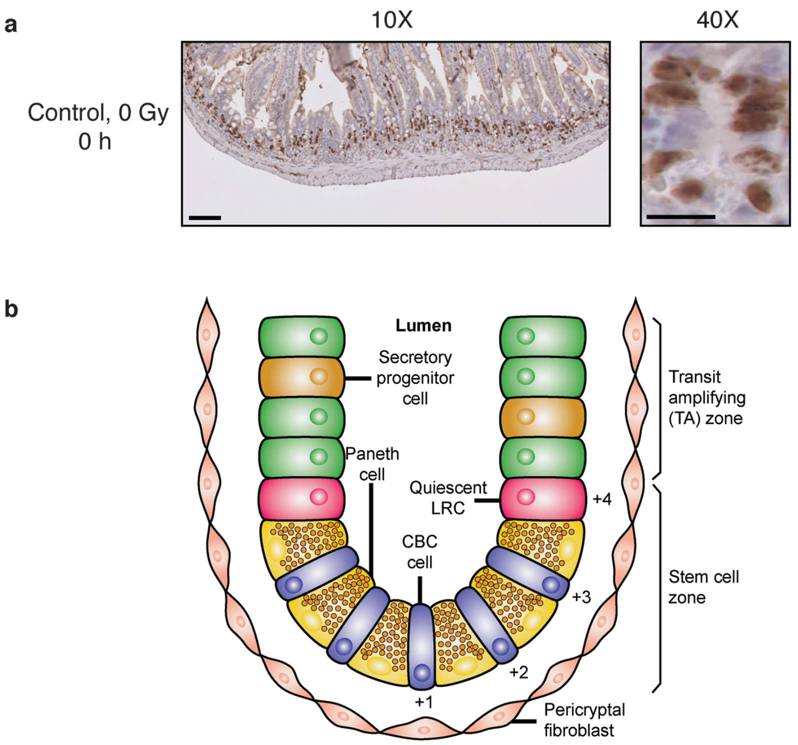

Supplementary Figure 3: BrdU staining of unirradiated intestinal crypt cells.
(a) Representative image of BrdU stained jejunum cross section at 10X magnification and the corresponding 40X magnified image of a crypt showing BrdU+ crypt cells/CBCs from unirradiated control animals. Scale bar shows 100 μm at 10X magnification and 25 μm at 40X magnification. **(b)** Schematic representation of the structure of the jejunal crypt with CBCs located at +1, +2 and +3 positions at the base of the crypt and sandwiched between Paneth cells. CBC=Crypt Base Columnar cell; LRC=Label Retaining Cell. The base of the crypt represents the stem cell zone and above this compartment is the transit amplifying (TA) zone. The TA compartment comprises TA cells with secretory progenitor cells interspersed between the TA cells, which matures into the Paneth cell population. Quantification of BrdU+ crypt cells, TUNEL+ crypt cells, and cleaved caspase-3+ crypt cells included cells from both the TA zone and the stem cell zone.

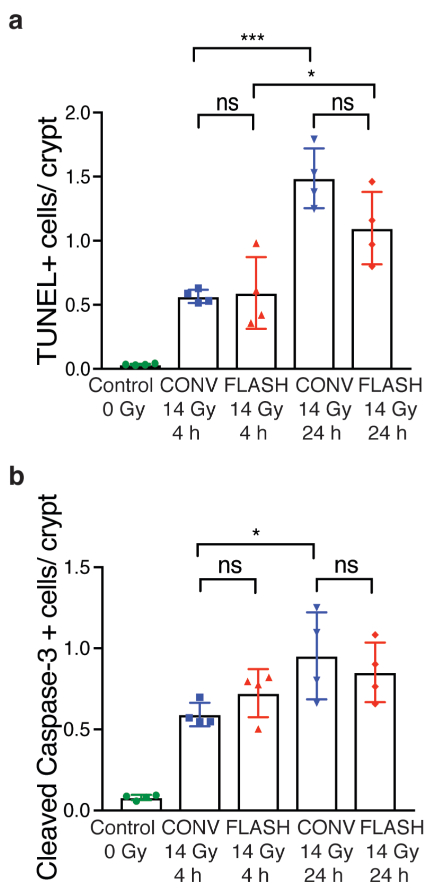

Supplementary Figure 4: Abdominal FLASH and CONV irradiation produce similar apoptosis of crypt cells in non-tumor bearing mice.

(a) Quantification of the average number of TUNEL+ cells per crypt and **(b)** average number of cleaved caspase-3+ cells per crypt in the jejunum analyzed at 4 hours (h) and 24 h after 14 Gy TAI, demonstrating no significant differences in apoptosis between FLASH and CONV irradiation when considering the entire crypt. TUNEL+ cells and cleaved caspase-3+ cells were quantified per crypt for crypts from 3 circumferences per mouse. n=4 mice per group. ns=no significant difference, *$p<0.05$, ***$p<0.001$. Comparisons by one-way ANOVA followed by Tukey's multiple comparisons test. Error bars represent standard deviation of the mean.
